# Unraveling the therapeutic mechanisms of dichloroacetic acid in lung cancer through integrated multi-omics approaches: metabolomics and transcriptomics

**DOI:** 10.3389/fgene.2023.1199566

**Published:** 2023-06-08

**Authors:** Malong Feng, Ji Wang, Jianying Zhou

**Affiliations:** ^1^ Department of Respiratory and Critical Care Medicine, The First Affiliated Hospital of Zhejiang University School of Medicine, Hangzhou, Zhejiang, China; ^2^ Department of Respiration, Fenghua District People’s Hospital of Ningbo, Ningbo, China; ^3^ Department of Infectious Diseases, Fenghua District People’s Hospital of Ningbo, Ningbo, China

**Keywords:** lung cancer, dichloroacetic acid (DCA), multi-omics, metabolomics, gene expression, molecular mechanisms, therapeutic target, drug mechanism

## Abstract

**Objective:** The aim of this study was to investigate the molecular mechanisms underlying the therapeutic effects of dichloroacetic acid (DCA) in lung cancer by integrating multi-omics approaches, as the current understanding of DCA’s role in cancer treatment remains insufficiently elucidated.

**Methods:** We conducted a comprehensive analysis of publicly available RNA-seq and metabolomic datasets and established a subcutaneous xenograft model of lung cancer in BALB/c nude mice (*n* = 5 per group) treated with DCA (50 mg/kg, administered via intraperitoneal injection). Metabolomic profiling, gene expression analysis, and metabolite-gene interaction pathway analysis were employed to identify key pathways and molecular players involved in the response to DCA treatment. *In vivo* evaluation of DCA treatment on tumor growth and MIF gene expression was performed in the xenograft model.

**Results:** Metabolomic profiling and gene expression analysis revealed significant alterations in metabolic pathways, including the Warburg effect and citric acid cycle, and identified the MIF gene as a potential therapeutic target in lung cancer. Our analysis indicated that DCA treatment led to a decrease in MIF gene expression and an increase in citric acid levels in the treatment group. Furthermore, we observed a potential interaction between citric acid and the MIF gene, suggesting a novel mechanism underlying the therapeutic effects of DCA in lung cancer.

**Conclusion:** This study underscores the importance of integrated omics approaches in deciphering the complex molecular mechanisms of DCA treatment in lung cancer. The identification of key metabolic pathways and the novel finding of citric acid elevation, together with its interaction with the MIF gene, provide promising directions for the development of targeted therapeutic strategies and improving clinical outcomes for lung cancer patients.

## Introduction

Lung cancer remains a formidable global health issue, accounting for a considerable percentage of cancer-related mortalities worldwide (Bunn, 2012). This complex and heterogeneous disease presents numerous challenges in terms of early diagnosis, treatment, and management. The late-stage detection of lung cancer often renders treatment less effective, emphasizing the need for improved diagnostic tools and screening methods to facilitate timely intervention (Hammerschmidt and Wirtz, 2009). Additionally, the high incidence of drug resistance and recurrence in lung cancer patients underscores the importance of developing novel therapeutic strategies. A comprehensive understanding of the molecular mechanisms underlying lung cancer is crucial to identifying innovative treatment options and overcoming the existing obstacles in lung cancer management (Mottaghitalab et al., 2019). Dichloroacetic acid (DCA), a small halogenated molecule, has recently emerged as a potential anti-cancer agent due to its ability to modulate cellular metabolism. The side effects and toxicities of DCA have been relatively well-documented. The most common side effects reported include peripheral neuropathy, which is reversible upon cessation of treatment, as well as liver enzyme abnormalities and gastrointestinal disturbances. In some cases, patients have experienced a mild and reversible cognitive decline. While these side effects are generally manageable, it is essential to balance the potential therapeutic benefits of DCA with the risk of adverse events (Farina et al., 2020). However, the precise mechanisms through which DCA exerts its therapeutic effects in lung cancer remain to be fully elucidated (Ma et al., 2018; Tataranni and Piccoli, 2019).

The utilization of multi-omics methodologies, incorporating metabolomics and transcriptomics, has demonstrated considerable potential in deciphering intricate biological systems and revealing previously unidentified therapeutic targets (Lefort et al., 2014). Metabolomics, the comprehensive analysis of endogenous small molecules within biological systems, can furnish valuable information on the metabolic ramifications of DCA administration. Concurrently, transcriptomics facilitates the examination of DCA-induced alterations in gene expression patterns. The amalgamation of these techniques can yield a more thorough understanding of DCA’s mode of action in the context of lung cancer treatment (Tataranni and Piccoli, 2019).

In the present investigation, our objective was to shed light on the therapeutic mechanisms of DCA in lung cancer by employing an integrated multi-omics approach, which encompasses both metabolomic and transcriptomic analyses. We utilized gas chromatography-time-of-flight mass spectrometry (GC-TOF-MS) to characterize the metabolomic profile of lung cancer cells subjected to DCA treatment, while RNA sequencing generated the corresponding transcriptomic data. Through the integration of these datasets, we endeavored to unravel the molecular pathways influenced by DCA and pinpoint potential biomarkers indicative of treatment response. Our findings may enhance the understanding of the molecular foundations of DCA’s therapeutic effects in lung cancer and offer invaluable insights for refining DCA-based treatment strategies. Moreover, the integrated multi-omics approach implemented in this research may serve as a template for subsequent inquiries into the mechanisms of other putative anti-cancer agents.

## Materials and methods

### Data collection and processing

We collected RNA-seq data from publicly available datasets. We extracted gene expression profiles from RNA-seq data in public datasets, including GSE10072, GSE12236, and GSE19188 (Lu et al., 2012; Zhang et al., 2018; Edginton-White et al., 2023). For transcriptomic data, we performed background correction, log2 transformation, and quantile normalization. For the metabolomic data, we applied missing value imputation, data transformation (log10), and autoscaling (mean-centering and dividing by the standard deviation) to obtain the normalized dataset. The primary purpose of using these datasets was to identify differentially expressed genes and potential biomarkers associated with DCA treatment in lung cancer. By integrating and analyzing the gene expression profiles from these datasets, we aimed to reveal the key molecular mechanisms underlying the therapeutic effects of DCA in lung cancer.

### Quantitative real-time PCR (qRT-PCR) analysis

Total RNA was extracted from tumor tissues using TRIzol reagent (Invitrogen, United States) following the manufacturer’s instructions. The quality of the extracted RNA was assessed using a NanoDrop spectrophotometer (Thermo Scientific, United States) and an Agilent 2100 Bioanalyzer (Agilent Technologies, United States). cDNA synthesis was performed using the RevertAid First Strand cDNA Synthesis Kit (Thermo Fisher Scientific, United States) according to the manufacturer’s instructions. Quantitative real-time PCR (qRT-PCR) was conducted using the PowerUp SYBR Green Master Mix (Thermo Fisher Scientific, United States) on a QuantStudio 6 Flex Real-Time PCR System (Applied Biosystems, United States). The primer sequences used in the study are as follows:

MIF Forward: 5′- GAA​CCG​CAA​CTA​CAG​TAA​GCT​GC -3′

MIF Reverse: 5′- ACG​TTG​GCA​GCG​TTC​ATG​TCG​T -3′

GAPDH Forward: 5′- CAT​CAC​TGC​CAC​CCA​GAA​GAC​TG -3′

GAPDH Reverse: 5′- ATG​CCA​GTG​AGC​TTC​CCG​TTC​AG -3′

The relative expression levels of the MIF gene were calculated using the 2^(-ΔΔCt) method, with the reference gene (GAPDH) serving as an internal control for normalization.

### Bioinformatics analysis

For the transcriptomic meta-analysis, we utilized the MetaIntegrator tool, a robust bioinformatics resource designed to integrate and analyze gene expression data from multiple studies (Haynes et al., 2017). The MetaIntegrator tool facilitated the identification of consistent gene expression signatures across diverse datasets, thereby enhancing the reliability of our findings. During the meta-analysis, both forward and backward searches were conducted to ensure a comprehensive assessment of the available data. Regarding the metabolomic analysis, we employed the MetaboAnalyst platform, a powerful and user-friendly web-based tool tailored for the interpretation of high-throughput metabolomics data (Chong et al., 2018). This platform enabled us to perform a series of advanced statistical analyses, including volcano plots to visualize the distribution of differentially expressed metabolites, principal component analysis (PCA) for dimensionality reduction and sample clustering, differential metabolite enrichment analysis to identify significantly altered metabolic features, and metabolic pathway analysis to investigate the biological functions and pathways impacted by DCA treatment. Additionally, we conducted metabolite-gene interaction analysis to explore the possible relationships between the identified metabolites and their corresponding genes, providing further insights into the molecular mechanisms underlying the therapeutic effects of DCA in lung cancer.

### Animal handling and treatment

We established a subcutaneous xenograft model of lung cancer using BALB/c nude mice, which were purchased from the Shanghai Laboratory Animal Center of the Chinese Academy of Sciences (Shanghai, China). The mice were housed in a temperature-controlled environment with a 12-h light/dark cycle and were provided with standard rodent chow and water *ad libitum*. After acclimatization, the animals were injected subcutaneously with A549 cells to establish the lung cancer model. Once the tumors were successfully established, the mice were divided into two experimental groups: the lung cancer control group and the lung cancer treatment group. In the lung cancer treatment group, the mice received dichloroacetic acid (DCA) at a concentration of 2 g/L (DCAC2) in their drinking water, while the lung cancer control group was administered an equivalent volume of 0.9% saline solution as their drinking water. At the end of the treatment period, the mice were euthanized following approved ethical guidelines, and serum samples were collected for further analyses.

### GC-TOF-MS

For the metabolomic profiling of mouse serum samples using gas chromatography-time-of-flight mass spectrometry (GC-TOF-MS), we adapted a published protocol with minor modifications to prepare and derivatize the samples. Initially, pooled quality control (QC) samples were created by combining 20 μL aliquots from each serum sample (Dunn et al., 2008). Subsequently, a 50 μL aliquot of serum sample was spiked with two internal standards (10 μL of L-2-chlorophenylalanine in water, 0.3 mg/mL; 10 μL of heptadecanoic acid in methanol, 1 mg/mL) and vortexed for 10 s. The mixed solution was extracted with 175 μL of methanol/chloroform (3:1) and vortexed for 30 s. After storing the samples for 10 min at −20°C, they were centrifuged at 8,000 rpm for 10 min. A 200 μL supernatant aliquot was transferred to a glass sampling vial and vacuum-dried at room temperature. The dried residue underwent a two-step derivatization process. First, 80 μL of methoxyamine (15 mg/mL in pyridine) was added to the vial, followed by incubation at 30°C for 90 min. Next, the samples were incubated with 80 μL of N,O-bis(trimethylsilyl)trifluoroacetamide (BSTFA, containing 1% trimethylchlorosilane (TMCS)) at 70°C for 60 min. Upon completion of the reaction, the samples were allowed to rest at room temperature for 1 h before proceeding with the GC-TOF-MS analysis.

### Cell culture

The human lung cancer cell line A549 was procured from the Cell Bank of the Chinese Academy of Sciences (Shanghai, China). Cells were maintained in Dulbecco’s Modified Eagle Medium (DMEM; Gibco, United States) supplemented with 10% fetal bovine serum (FBS; Gibco, United States) and 1% penicillin-streptomycin (Gibco, United States). Cultures were incubated at 37°C in a humidified atmosphere containing 5% CO_2_.

## Results

### Metabolomic profiling

**FIGURE 1:**
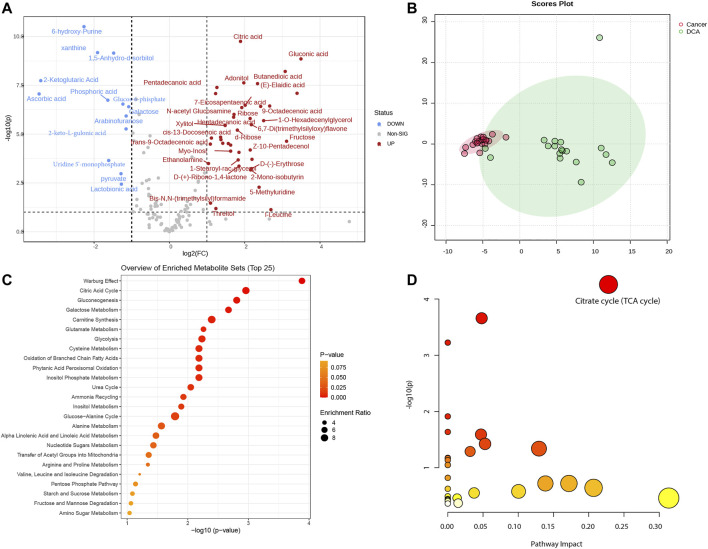
Metabolomic analysis of lung cancer control and DCA treatment groups. **(A)** Volcano plot displaying the differentially expressed metabolites between the lung cancer control group and the DCA treatment group. Red dots represent upregulated metabolites, while blue dots indicate downregulated metabolites. The threshold for differential expression is set at |log2(Fold change)|> 1 and -log10(P) > 1. **(B)** Principal component analysis (PCA) of the differentially expressed metabolites, showing a clear separation between the lung cancer control group (red) and the DCA treatment group (green). **(C)** Pathway enrichment analysis of the differentially expressed metabolites, with the Warburg effect and Citric acid cycle emerging as the most significantly enriched pathways. **(D)** Pathway impact analysis, revealing the Citrate cycle (TCA cycle) as the most significantly impacted pathway in the DCA treatment group.

In this study, we utilized a total of 38 mice, comprising 18 in the DCA treatment group and 20 in the lung cancer control group. Based on our previously defined criteria for selecting differentially expressed metabolites, we set the threshold at |log2(Fold Change)|> 1 and -log10P) > 1. A total of 53 differentially expressed metabolites were identified between the two groups (see Supplementary Table S1 for details). These metabolites were visualized using a volcano plot to demonstrate their differential expression (Figure 1A). We conducted a principal component analysis (PCA) on the differentially expressed metabolites between the lung cancer and DCA treatment groups. The PCA revealed a significant separation between the two groups (Figure 1B), indicating distinct metabolic profiles. Subsequently, we performed pathway enrichment analysis and pathway impact analysis on the differentially expressed metabolites. The pathway enrichment analysis revealed that the two most significantly enriched pathways were the Warburg effect and the Citric acid cycle (Figure 1C). In the pathway impact analysis, the Citrate cycle (TCA cycle) emerged as the most significantly impacted pathway (Figure 1D), suggesting a potential influence of DCA treatment on these metabolic processes in lung cancer.

### Gene expression profiles in lung cancer

**FIGURE 2:**
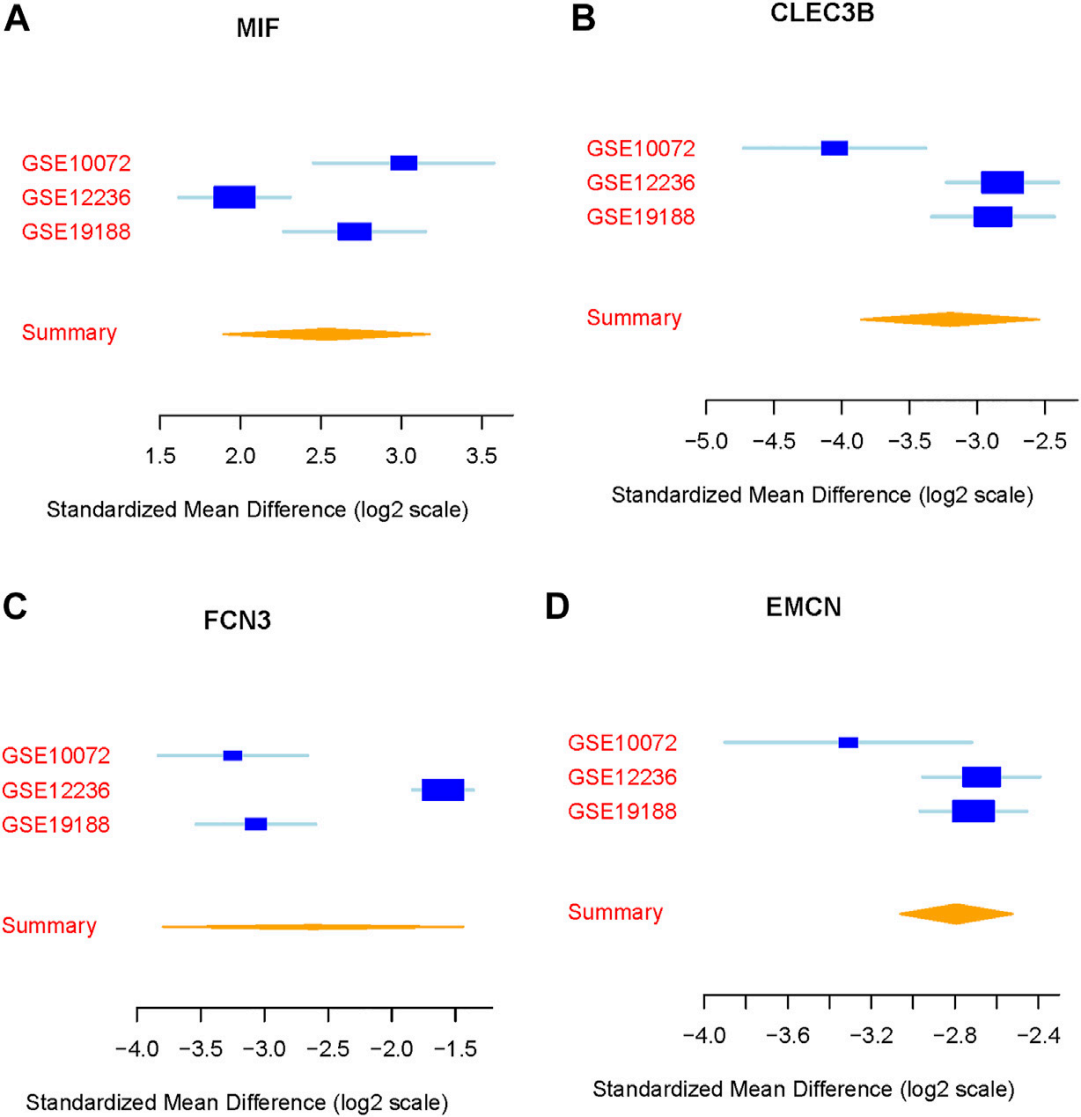
Identification of differentially expressed genes in lung cancer. **(A–D)** Expression patterns of the four highly significant differentially expressed genes (MIF, CLEC3B, FCN3, and EMCN) across the three public datasets GSE10072, GSE12236, and GSE19188.

**FIGURE 3:**
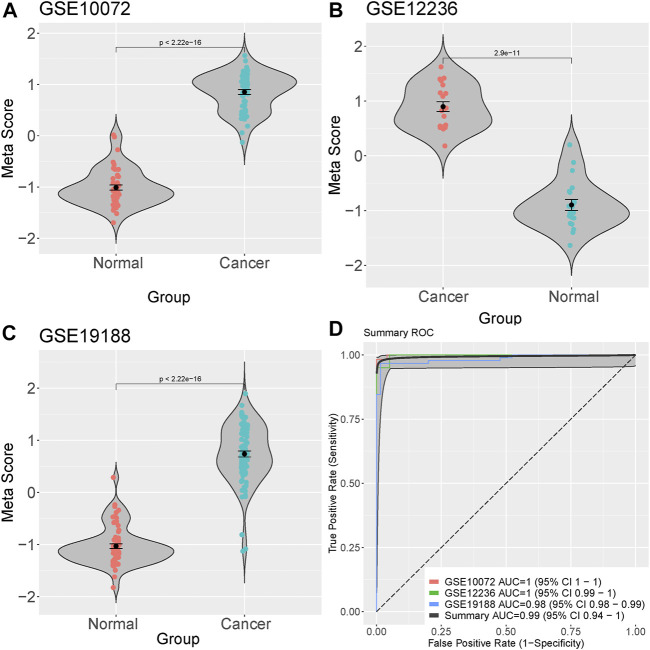
Diagnostic potential of the four identified genes in Lung cacner. **(A–C)** Meta-score generation by combining the expression levels of the four differentially expressed genes (MIF, CLEC3B, FCN3, and EMCN) in each sample. **(D)** Summary receiver operating characteristic (ROC) curve analysis demonstrating the diagnostic accuracy of the meta-score, with an area under the curve (AUC) of 0.99.

We integrated and analyzed RNA-seq data from three public datasets, including GSE10072, GSE12236, and GSE19188. Our analysis identified four highly significant differentially expressed genes in lung cancer, comprising MIF, CLEC3B, FCN3, and EMCN (Figures 2A–D). The consistent validation of these genes across multiple datasets suggests their potential importance in lung cancer. To further investigate the diagnostic potential of these four genes, we generated a meta-score by combining their expression levels in each sample (Figures 3A–C). We then used this meta-score to distinguish between lung cancer and normal control samples. The summary receiver operating characteristic (ROC) curve analysis revealed that the area under the curve (AUC) was 0.99, indicating high diagnostic accuracy for lung cancer (Figure 3D).

### Metabolite-metabolite and metabolite-gene interaction pathway analysis

**FIGURE 4:**
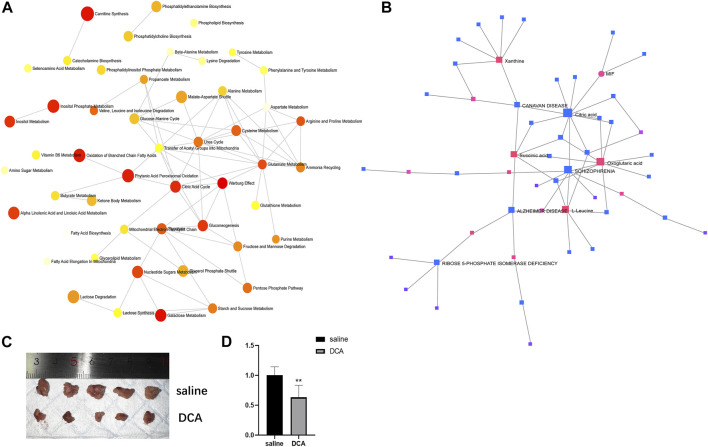
Interactions between metabolites and pathways, and *in vivo* evaluation of DCA treatment. **(A)** Network analysis of differentially expressed metabolites showing interactions between the Warburg effect and Citric acid cycle pathways. **(B)** Interaction between citric acid and MIF gene in lung cancer. **(C)** Representative images of subcutaneous xenograft tumors in the cancer control and DCA treatment groups, showing a significant difference in tumor size. **(D)** RT-PCR analysis of MIF gene expression in tumors from the cancer control and DCA treatment groups, demonstrating a significant reduction in MIF expression in the DCA-treated group.

In order to explore the relationships between differentially expressed metabolites and their associated genes, as well as the interactions among the metabolites themselves, we conducted a series of analyses. First, we performed a metabolite-pathway interaction analysis on the 53 differentially expressed metabolites. In the resulting network, we observed interactions between the Warburg effect and Citric acid cycle pathways (Figure 4A). Subsequently, we investigated the interactions between metabolites and genes. Our analysis revealed a significant interaction between the core metabolite citric acid, which is involved in the Citric acid cycle, and the differentially expressed gene MIF in lung cancer (Figure 4B). This finding suggests a potential link between the identified metabolites and genes in the context of lung cancer pathogenesis.

### *In vivo* evaluation of DCA treatment on tumor growth and MIF gene expression

To assess the impact of DCA treatment on tumor growth *in vivo*, we compared the subcutaneous xenograft tumors in the cancer control group and the DCA treatment group. As depicted in Figure 4C, there was a significant difference in tumor size between the two groups, with the DCA treatment group exhibiting notably smaller tumors compared to the cancer control group, which received saline. We then performed RT-PCR analysis to examine the expression of the MIF gene in the tumors of both groups. The results, shown in Figure 4D, revealed a significant decrease in MIF gene expression in the DCA treatment group compared to the cancer control group. These findings suggest that DCA treatment may effectively suppress tumor growth and modulate MIF gene expression in the lung cancer xenograft model.

## Discussion

In this study, we aimed to explore the effects of DCA treatment on lung cancer by integrating transcriptomic and metabolomic data, as well as validating our findings using an *in vivo* lung cancer xenograft model. Our comprehensive analysis not only provided insights into the molecular mechanisms underlying the therapeutic effects of DCA in lung cancer but also identified potential diagnostic biomarkers and therapeutic targets.

We found 53 differentially expressed metabolites between the DCA treatment and lung cancer control groups. Our pathway enrichment and impact analyses revealed that the Warburg effect and Citric acid cycle were the most significantly enriched and impacted metabolic pathways, respectively. Importantly, we observed a significant increase in citric acid levels in the DCA treatment group. Previous studies have reported that elevated citric acid can inhibit the growth of A549 cells (Zhou et al., 2015; Ji et al., 2020). Furthermore, our analysis indicated a potential interaction between citric acid and the MIF gene. These findings suggest a novel mechanism by which DCA may exert its anticancer effects, involving the modulation of citric acid levels and its subsequent interaction with the MIF gene.

The Warburg effect, a well-known metabolic alteration in cancer cells, is characterized by an increased rate of glycolysis even under nonmonic conditions, leading to lactate production instead of oxidative phosphorylation in the mitochondria (Liberti and Locasale, 2016). Our findings suggest that DCA may exert its anticancer effects by targeting these key metabolic pathways and shifting the cancer cells’ metabolism away from the Warburg effect towards oxidative phosphorylation, which ultimately leads to increased ROS production and subsequent cell death. This is in line with previous studies demonstrating that DCA can reverse the Warburg effect in cancer cells and promote apoptosis via the mitochondria-dependent pathway.

Our analysis also identified four highly significant differentially expressed genes in lung cancer, namely, MIF, CLEC3B, FCN3, and EMCN. Among these, MIF was found to interact significantly with the core metabolite citric acid in the Citric acid cycle. MIF, also known as macrophage migration inhibitory factor, is a pleiotropic cytokine implicated in various biological processes, including cell proliferation, angiogenesis, and immune regulation. Overexpression of MIF has been reported in several cancer types, including lung cancer, and is associated with tumor progression, metastasis, and poor prognosis (Verjans et al., 2009; Nobre et al., 2017; Penticuff et al., 2019). Our findings suggest that the therapeutic effects of DCA in lung cancer may be partially mediated through the regulation of MIF expression and its interaction with citric acid. Further investigation of MIF as a potential therapeutic target in lung cancer is warranted. Besides lung cancer, DCA has been investigated in numerous other cancer types, including breast cancer, glioblastoma, colorectal cancer, and prostate cancer. In addition to the Warburg effect reversal, DCA treatment has been shown to modulate other signaling pathways and cellular processes in various cancer types. For instance, in breast cancer, DCA has been found to inhibit the Akt/mTOR signaling pathway, leading to the suppression of cell proliferation and migration (Xiao et al., 2017). In glioblastoma, DCA has been reported to enhance the activity of the DNA repair enzyme O6-methylguanine-DNA methyltransferase (MGMT), thereby increasing the sensitivity of glioblastoma cells to temozolomide, a standard chemotherapeutic agent (Singh et al., 2021). In colorectal cancer, DCA has been shown to modulate the p53 signaling pathway, promoting cell cycle arrest and apoptosis (Zeng et al., 2015).

In our *in vivo* lung cancer xenograft model, we observed that DCA treatment significantly reduced tumor size and suppressed MIF gene expression. This corroborates our *in silico* findings and provides evidence for the potential therapeutic value of DCA in lung cancer treatment. Although DCA has been widely studied for its anticancer properties, clinical trials involving DCA for cancer treatment have yielded mixed results. Our study adds to the growing body of evidence supporting the potential of DCA as a therapeutic agent in lung cancer and provides a rationale for further investigation into the optimal dosing, treatment duration, and possible combination therapies with other anticancer agents to enhance its efficacy and minimize potential side effects.

In summary, our integrated transcriptomic and metabolomic analysis, together with *in vivo* validation, provided valuable insights into the molecular mechanisms underlying the therapeutic effects of DCA in lung cancer. We identified key metabolic pathways, including the novel finding of citric acid elevation and its interaction with the MIF gene, potential diagnostic biomarkers, as well as therapeutic targets, which may help guide future research and clinical management of lung cancer. Nevertheless, further studies with larger sample sizes and diverse cancer models are needed to confirm our findings and establish the clinical utility of DCA in the treatment of lung cancer. Additionally, investigations into the potential synergistic effects of DCA in combination with other anticancer agents may help optimize its therapeutic potential and overcome potential resistance mechanisms.

In conclusion, our study highlights the importance of integrated omics approaches in unraveling the complex molecular mechanisms underpinning the therapeutic effects of DCA in lung cancer. The identification of key metabolic pathways, including the novel finding of citric acid elevation and its interaction with the MIF gene, offers promising avenues for the development of targeted therapeutic strategies and the improvement of clinical outcomes for lung cancer patients.

## Data Availability

The original contributions presented in the study are included in the article/Supplementary Materials, further inquiries can be directed to the corresponding author.
